# Hypoxia Modulates the Response of Mast Cells to *Staphylococcus aureus* Infection

**DOI:** 10.3389/fimmu.2017.00541

**Published:** 2017-05-11

**Authors:** Helene Möllerherm, Katja Branitzki-Heinemann, Graham Brogden, Ayssar A. Elamin, Wulf Oehlmann, Herbert Fuhrmann, Mahavir Singh, Hassan Y. Naim, Maren von Köckritz-Blickwede

**Affiliations:** ^1^Department of Physiological Chemistry, University for Veterinary Medicine Hannover, Hanover, Germany; ^2^LIONEX Diagnostics & Therapeutics, Braunschweig, Germany; ^3^Faculty of Veterinary Medicine, Institute of Biochemistry, University of Leipzig, Leipzig, Germany; ^4^Research Center for Emerging Infections and Zoonoses (RIZ), University for Veterinary Medicine Hannover, Hanover, Germany

**Keywords:** mast cells, innate immunity, extracellular traps, degranulation, hypoxia, hypoxia-inducible factor 1α

## Abstract

To study the antimicrobial function of immune cells *ex vivo*, cells are commonly cultivated under atmospheric oxygen concentrations (20–21%; normoxia), although the physiological oxygen conditions *in vivo* are significantly lower in most tissues. Especially during an acute infection, oxygen concentration locally decreases to hypoxic levels around or below 1%. The goal of this study was to investigate the effect of hypoxia on the activity of mast cells (MCs). MCs were cultivated for 3 or 24 h at 1% O_2_ in a hypoxia glove box and co-incubated with heat-inactivated *Staphylococcus aureus*. When incubating the cells for 24 h under hypoxia, the transcriptional regulator hypoxia-inducible factor 1α (HIF-1α) was stabilized and resulted in increased extracellular trap formation and decreased phagocytosis. Interestingly, while phagocytosis of fluorescent *S. aureus* bioparticles as well as the release of extracellular traps remained unaffected at 3 h hypoxia, the secretion of the prestored mediator histamine was increased under hypoxia alone. In contrast, the release of TNF-α was generally reduced at 3 h hypoxia. Microarray transcriptome analysis revealed 13 genes that were significantly downregulated in MCs comparing 3 h hypoxia versus normoxia. One interesting candidate is *sec24*, a member of the pre-budding complex of coat protein complex II (COPII), which is responsible for the anterograde transport of proteins from the ER to the Golgi apparatus. These data lead to the suggestion that *de novo* synthesized proteins including crucial factors, which are involved in the response to an acute infection like TNF-α, may eventually be retained in the ER under hypoxia. Importantly, the expression of HIF-1α was not altered at 3 h. Thus, our data exhibit a HIF-1α-independent reaction of MCs to short-term hypoxia. We hypothesize that MCs respond to short-term low oxygen levels in a HIF-1α-independent manner by downregulating the release of proinflammatory cytokines like TNF-α, thereby avoiding uncontrolled degranulation, which could lead to excessive inflammation and severe tissue damage.

## Introduction

Although best known for their role as key mediators in the early and acute allergic reactions as well as for their activation during certain parasitic infections (1), mast cells (MCs) also play an important protective role against various microbial infections, e.g., the Gram-positive pathogen *Staphylococcus* (*S*.) *aureus* (2, 3). Recently, they have attracted increasing attention as key immunomodulatory cells and represent themselves, moreover, as tissue-specific multifunctional, sentinel cells of the innate immune system (4).

Mast cells are activated by several stimuli: numerous receptors on the surface facilitate MCs for early and rapid sensing of invading microorganisms such as bacteria, parasites, fungi, and viruses (5). On one hand, they can act directly antimicrobial: MCs are able to phagocyte a broad spectrum of bacteria, e.g., *S. aureus, Streptococcus faecium, Klebsiella pneumoniae, Citrobacter freundii*, and *Escherichia coli* (3, 6) involving the intracellular endosome–lysosome pathway, in which the bacteria are killed through a combination of oxidative and non-oxidative killing (3, 7). Moreover, MCs have also been shown to release their nuclear DNA and subsequently form antimicrobial MC extracellular traps (MCETs) to immobilize and kill pathogens, similar to neutrophil extracellular traps (NETs) (2).

Even more important, MCs are well known to orchestrate the immune response by releasing various mediators (8). These long-living sentinel cells are crucial for the early recruitment of effector cells, e.g., professional phagocytes like neutrophils to the local infection (9), and thereby set the stage for an appropriate acquired immune response. MCs are able to selectively release various mediators like TNF-α and histamine within seconds after recognition of a pathogen (9). This short-term response is facilitated by the prestoring of histamine and TNF-α in their granules (10, 11).

During an inflammation or immune response to an infection, immune cells infiltrate and locally consume oxygen. In addition, invading bacteria like *S. aureus* have an oxygen-depleting impact (12). Since oxygen is an elementary component of the microenvironment required for cell activity (13), the question arises, whether immune cells like MCs as key modulators of the host defense reaction respond differently under hypoxia in infected tissues. However, in most tissue culture experiments, cells are cultivated under normoxic conditions, ignoring the fact that immune cells normally function under hypoxia in inflamed tissue (0.1–3% O_2_) (14). For human neutrophils, it was already shown that hypoxia enhanced bactericidal activities (15), increased their chemotactic, phagocytic and respiratory burst, but led to a decrease in NET formation (16) and protected them from apoptosis (17–20).

Hypoxia-inducible factor 1α (HIF-1α) is well known to play an important role in the adaptation to oxygen shortage (21–24). This transcription factor is widely expressed in a variety of cell types including macrophages (25), neutrophils (26), and T-cells (27). HIF-1α expression is regulated on protein level and mediates central functions of macrophages and neutrophils in innate host defense (28). During normoxia, in the presence of oxygen, HIF-1α is proteasomally degraded. Conversely, during hypoxia, HIF-1α is stabilized and is in charge of an adaptive transcriptional response (29). It has been shown that pharmacologically stabilized HIF-1α levels, even under normoxia, mediate the extracellular antimicrobial activity of human and murine MCs by increasing the formation of MCETs (30). However, the effect of oxygen shortage on MC functionality as response to a bacterial infection has not been fully addressed yet and is the goal of this study.

## Materials and Methods

### Bone Marrow-Derived MC (BMMC) Isolation

Bone-marrow-derived MCs from C57BL/6 wild-type (WT) mice were isolated and cultured over 4 weeks in T25 suspension culture flasks (Sarstedt) in the presence of IL-3 (10 ng/ml) as previously described (2). To determine the purity and differentiation status of BMMCs, cells were stained with a phycoerythrin (PE)-labeled anti-mouse CD117 antibody (0.06 μg/10^6^ cells) (Biolegend) and analyzed by flow cytometry using an Attune NxT Flow Cytometer (Thermo Fisher Scientific). MCs were used for experiments if more than 95% of the cells were confirmed to be CD117 positive.

### Bacterial Strains

The bacterial strains used in this study were the well characterized and widely used laboratory strain *S. aureus* Newman and the clinical community-acquired MRSA strain USA 300 (LAC AH 1263). Both strains were grown in brain heart infusion (BHI) medium at 37°C with shaking. Fresh overnight cultures were diluted 1:50 in BHI and then grown to mid-exponential growth phase (OD_600_ = 0.7) until usage. Heat inactivation (h.i.) was performed for 30 min in 95°C hot water in a volume of 50 ml (*S. aureus* USA 300) and 5 min in 95°C heating block in a volume of 500 μl (*S. aureus* Newman). The efficiency of heat inactivation was proven by plating on blood agar plates.

### Oxygen Measurement

Oxygen measurements were performed as previously described (31) using a Fibox4-PSt3 measurement system (PreSens Precision Sensing GmbH) in 24-well plates (Nunc, Germany). Importantly, oxygen was measured non-invasively and was not consumed during the process of measurement. Using optical sensors (placed on the bottom of the wells in the medium), the dissolved oxygen level in the cell culture media was measured based on the oxygen-dependent quenching of phosphorescent probes (16, 31, 32). Oxygen measurements were performed over a time period of 5 h while the cells were incubated under hypoxic (7 mmHg, 1% O_2_; 5% CO_2_) or normoxic (159 mmHg, 21% O_2_; 5% CO_2_) conditions, respectively.

### Western Blot Analysis of HIF-1α and Sec24A Protein Levels

For the western blot analysis of HIF-1α, BMMCs in a density of 1 × 10^6^ cells/ml in a volume of 3 ml IMDM (supplemented with 0.1 mM MEM and 2% of 70°C h.i. FCS) were incubated 3 and 24 h under hypoxia (7 mmHg, 1% O_2_; 5% CO_2_) or, respectively, under normoxia (159 mmHg, 21% O_2_; 5% CO_2_) in a 35 mm × 10 mm cell culture dish (Sarstedt). For the western blot analysis of Sec24A, BMMCs in a density of 1 × 10^6^ cells/ml in a volume of 1 ml IMDM (supplemented with 0.1 mM MEM and 2% of 70°C h.i. FCS) were incubated 3 h under hypoxia (7 mmHg, 1% O_2_; 5% CO_2_) or respectively under normoxia (159 mmHg, 21% O_2_; 5% CO_2_) in a 1.5 ml reaction tube. Then, the cells were transferred and centrifuged at 180 *g* at 4°C for 5 min to remove the supernatant. The pellet was resuspended in 65 μl of lysis buffer [10 mM Tris (pH 7.9) 2 mM EDTA, 150 mM NaCl, 0.5% NP40 (Igepal)] containing 1:5 proteinase inhibitors [1.48 μM antipain dihydrochloride, 0.768 μM aprotinin, 10.51 μM leupeptin, 1.46 μM pepstatin A, 50 μgml^−1^ trypsin-inhibitor, 1 mM phenylmethanesulfonyl fluoride; Sigma] and incubated for 20 min at 4°C. After centrifugation at 4,500 *g* at 4°C for 5 min, the supernatant was harvested and the amount of protein was determined by Bradford. Seventy-five micrograms for HIF-1α and 50 μg for Sec24A of total cell extracts were denaturized in Laemmli buffer [150 mM Tris/HCl (pH 6.8), 6% SDS, 30% glycerin, and 0.02% bromophenol blue] for 5 min at 95°C. Samples were separated by SDS/PAGE (10% gels) and then proteins were transferred to nitrocellulose membranes for HIF-1α and PVDF for Sec24A. The protein levels and β-actin protein were detected by incubating the membrane with respective primary antibodies against HIF-1α (rabbit anti-HIF-1α, GeneTex, 2 μg/ml) and Sec24A (rabbit anti- Sec24A, Proteintech, 0.2 μg/ml) overnight at 4°C and β-actin (mouse anti-β-actin, Santa Cruz Biotechnology, 0.02 μg/ml) 45 min at room temperature. After washing, membranes were incubated for 45 min at room temperature with the respective secondary antibodies: horseradish peroxidase-conjugated goat anti-rabbit IgG (Thermo Scientific) for HIF-1α and Sec24A and goat anti-mouse IgG (Thermo Scientific) for β-actin. After washing, protein was visualized using the SuperSignal West Femto Chemiluminescent Substrate reagents (Pierce, Thermo Scientific).

### MCET Visualization and Determination of Viability of MCs under Hypoxia

BMMCs were seeded in 96-well glass bottom plates (MatTec) in a density of 7 × 10^4^ BMMCs in 100 μl (supplemented with 0.1 mM MEM) per well and incubated 3 or 24 h under hypoxia (7 mmHg, 1% O_2_; 5% CO_2_) or normoxia (159 mmHg, 21% O_2_; 5% CO_2_). LIVE/DEAD viability/cytotoxicity kit for mammalian cells (Invitrogen) was used to determine MCET-releasing cells and the viability of MCs without fixation following the recommendations of the manufacturer. Cells were analyzed by confocal fluorescence microscopy using a Leica TCS SP5 confocal microscope with a HCX PL APO 40× 0.75–1.25 oil immersion objective. Results are shown from the analysis of three independent experiments, each with four individual images (min. 100 cells per slide).

### Phagocytosis of *S. aureus* Bioparticles

A total of 5 × 10^5^ BMMCs in 250 μl IMDM (supplemented with 0.1 mM MEM and 2% of 70°C h.i. FCS) were preincubated 3 or 24 h under hypoxia (7 mmHg, 1% O_2_; 5% CO_2_) or, respectively, under normoxia (159 mmHg, 21% O_2_; 5% CO_2_) in a 5 ml, 75 mm × 12 mm tube. After the incubation, PE-labeled *S. aureus* (Wood strain, bioparticles; Sigma) at an MOI of 60 was added for 30 min. The reaction was stopped on ice and MCs were washed with PBS and centrifuged at 90 *g* for 10 min/4°C to remove non-associated bacteria. PE fluorescence was measured using a Beckman Coulter EPICS XL flow cytometer. The red fluorescence intensity per MC (% gated) was recorded and represents the mean relative phagocytosis of PE-labeled *S. aureus* per MC.

### TNF-α Determination in Cell Culture Supernatants

Supernatants were collected from a total of 1 × 10^6^ cells/ml. BMMCs were incubated in a 1.5 ml reaction tube in a volume of 900 μl IMDM (supplemented with 0.1 mM MEM and 2% of 70°C h.i. FCS) for 3 h at either under hypoxia (7 mmHg, 1% O_2_; 5% CO_2_) or normoxia (159 mmHg, 21% O_2_; 5% CO_2_). After 3 h, BMMCs were infected with an MOI 1 with h.i. *S. aureus* Newman and h.i. *S. aureus* USA 300 for 45 min. The reaction was stopped on ice, BMMCs were centrifuged 180 *g*/4°C for 5 min. Supernatants were collected and frozen in liquid nitrogen. The TNF-α ELISA (Murine TNF-α ELISA development kit, PeproTech) was performed following the manufacturer’s instructions in triplicates.

### Histamine Determination in Cell Culture Supernatants

For studies of histamine content and release, BMMCs were washed three times in calcium magnesium-free HBSS and then suspended in complete HBSS containing 25 mM HEPES and 0.1% BSA. A total of 1 × 10^6^ BMMCs were incubated in a 1.5 ml reaction tube for 3 h either under hypoxia (7 mmHg, 1% O_2_; 5% CO_2_) or normoxia (159 mmHg, 21% O_2_; 5% CO_2_). After 3 h, BMMCs were incubated with an MOI 1 h.i. *S. aureus* Newman and h.i. *S. aureus* USA 300 in a volume of 100 μl, stimulated with the MC-degranulating peptide mastoparan (50 μM; Bachem, Heidelberg, Germany) as a positive control and HBSS (spontaneous release) for 45 min. The reaction was stopped on ice, BMMCs were centrifuged 450 *g* at 4°C for 10 min. Supernatants were separated and the cell pellet was resuspended in 950 μl PBS. The histamine release was measured by rpHPLC as described earlier (33).

### Lipid Isolation and Analysis

A total of 3 × 10^6^ BMMCs were incubated in a 1.5 ml reaction tube in 1 ml IMDM (supplemented with 0.1 mM MEM and 2% of 70°C h.i. FCS) for 3 h either under hypoxia (7 mmHg, 1% O_2_; 5% CO_2_) or normoxia (159 mmHg, 21% O_2_; 5% CO_2_). Samples were washed twice with PBS, resuspended in chloroform–methanol (1:1), and lysed by passing cells through a 45-mm cannula syringe 15 times. Subsequent lipid isolation was performed as previously described (16, 34).

Cholesterol content was analyzed with a Hitachi Chromaster HPLC using a Chromolith^®^ HighResolution RP-18 endcapped 100–4.6 mm column coupled to a 5–4.6 mm guard cartridge and heated to 32°C. Methanol was used as the mobile phase at a flow rate of 1 ml/min at 60 bar, and a UV detector measuring at 202 nm to determine the amount of cholesterol in each sample. The results were quantified against an external standard ranging from 0.05 to 2 mg/ml cholesterol and expressed as nanogram cholesterol per 1 × 10^6^ MCs.

Triglycerides, free fatty acids (FFAs), monoacylglycerols (MGs), and phospholipids were analyzed by high performance thin layer chromatography (HPTLC) based on a method described previously (16, 34). Briefly, isolated lipid samples were loaded on high performance silica gel plates (Merck, Germany) and separated based on polarity. Lipids were visualized by copper sulfate solution and the band intensities subsequently analyzed by CP Atlas (Lazer Software). Lipids were identified against a known standard. Each sample was analyzed in repetition.

### RNA Expression Analysis

RNA was extracted from 1 × 10^6^ BMMCs after incubation under normoxia or hypoxia for 3 h in IMDM (supplemented with 0.1 mM MEM and 2% of 70°C h.i. FCS) in a 1.5-ml reaction tube, with the RNeasy Mini Kit (Qiagen) as described in the user’s manual. RNA quality was tested with a bioanalyzer (RNA 6000 Pico Kit, Agilent) following the manufacturer’s instructions. The RNA quality was scored based on RNA integrity (RIN), a numbering system from 1 to 10, with 1 being the most degraded profile and 10 being the most intact (35). A RIN number of ≥8.5 was considered as good quality. Real-time PCR of reverse transcribed RNA (RT-qPCR) was designed to analyze expression of genes of interest and the housekeeping gene *rps9*. The respective primers are given in Table 1. The RT-qPCR was conducted as previously described (16) with the following modified program: initial denaturation at 95°C for 20 min and 40 cycles of denaturation at 95°C for 25 s, annealing at 64°C for 30 s, and amplification at 72°C for 20 s using an AriaMX Real-Time PCR system. Products were verified by melting curve analysis and 1% agarose gel electrophoresis. Data were normalized to a non-regulated housekeeping gene (*rps9*). The relative ΔCT values were determined for expression of *hif-1α* and *tnf-α*. CT is the cycle number at the chosen amplification threshold, ΔCT = CT gene (*tnf-α*) − CT reference (*rsp9*) and ΔΔCT = ΔCT sample − ΔCT calibrator. The fold change in expression (2^−ΔΔCT^) was calculated as the read-out parameter. The calibrator was MCs under normoxia.

**Table 1:** **Oligonucleotide primers used in RT-qPCR**.

Primer	Accession number	Sequence (sense, antisense)	RNA/DNA	Tm°C
Rps9	NM_029767	Forward primer TTGTCGCAAAACCTATGTGACC	147/344	61.1
		Reverse primer GCCGCCTTACGGATCTTGG		62.8
TNF-α	X02611	Forward primer CCTGTAGCCCACGTCGTAG	148/442	61.5
		Reverse primer GGGAGTAGACAAGGTACAACCC		61.4
HIF-1α	NM_001313919.1	Forward primer CATCCAGAAGTTTTCTCACACG	138/–	63.5
		Reverse primer GGCGAAGCAAAGAGTCTGAA		64.5

### RNA and Array Processing

RNA was extracted from 6 × 10^5^ BMMCs incubated 3 h under hypoxia (7 mmHg, 1% O_2_; 5% CO_2_) or normoxia (159 mmHg, 21% O_2_; 5% CO_2_) in a 1.5 ml reaction tube.

The RNA samples were processed using the GeneChip^®^ WT Plus Reagent Kit (Affymetrix, Cat.-No. 902280) according to the manufacturer’s protocol. In brief, the total RNA was first transcribed to double stranded cDNA, which was transcribed to cRNA. The cRNA was then synthesized into single stranded cDNA, which was fragmented and biotinylated. Finally, the biotinylated single stranded cDNA was hybridized onto the whole transcript Affymetrix^®^ Mouse Gene 2.1 ST arrays (Affymetrix, Cat.-No. 902120), which covers a total of 35, 240 transcripts. After staining with a streptavidin phycoerythrin conjugate, the strips were washed and by the Washing Station and finally scanned the Imaging Station of the GeneAtlas^®^System (Affymetrix) using GeneAtlas HWS Kit for WT Arrays (Affymetrix, Cat.-No. 901667). These steps were carried out following GeneAtlas user guide. The probe cell intensity data (CEL) files were generated using the Command Console™ software (Affymetrix).

### Gene and Exon Expression Analysis

The CEL files were imported into Partek Genomics Suite version 6.6 (Partek Inc., St. Louis, MO, USA) for the gene and exon expression analysis using the robust multi-array average settings for normalization. Differential expression was examined using the 1-way ANOVA statistic with a significance cutoff of *p* < 0.05 and a fold change >1.3. The statistical robustness of the expression data was visualized with principle component analysis (PCA), provided within the Partek Genomic Suite 6.6 software (36–42). The quality of the experiments was assessed on the basis of the QC metrics table and QC graphical report. Spearman’s correlation was used as a similarity matrix to conduct average linkage hierarchical clustering. Differentially expressed genes were grouped into functional categories ranked according to their *p*-values using the Partek Gene Ontology Enrichment tool.

### Pathway Analysis

The fold-change filtered differentially expressed genes were considered further and the functions of the genes explored using Pathway analysis utilizing KEGG database (Partek^®^ Pathway™). Partek Pathway was used to determine and visualize significantly enriched pathways (using a Fisher’s exact test). The top enriched Partek pathways for the differentially expressed genes were sorted by the Enrichment Score (Fisher’s exact test).

### Statistical Analysis

Three independent experiments were performed at least unless indicated otherwise. Data were analyzed using Excel 2010 (Microsoft) and GraphPad Prism 7.0 (GraphPad Software). Differences between two groups were analyzed by using an unpaired, two-tailed Student’s *t*-test, if not otherwise stated. The significance is indicated as follows: ns, not significant, *p ≤ 0.05, **p ≤ 0.01, ***p ≤ 0.001, and ****p < 0.0001.

## Results

### Viability of MCs under Hypoxia

**Figure 1:**
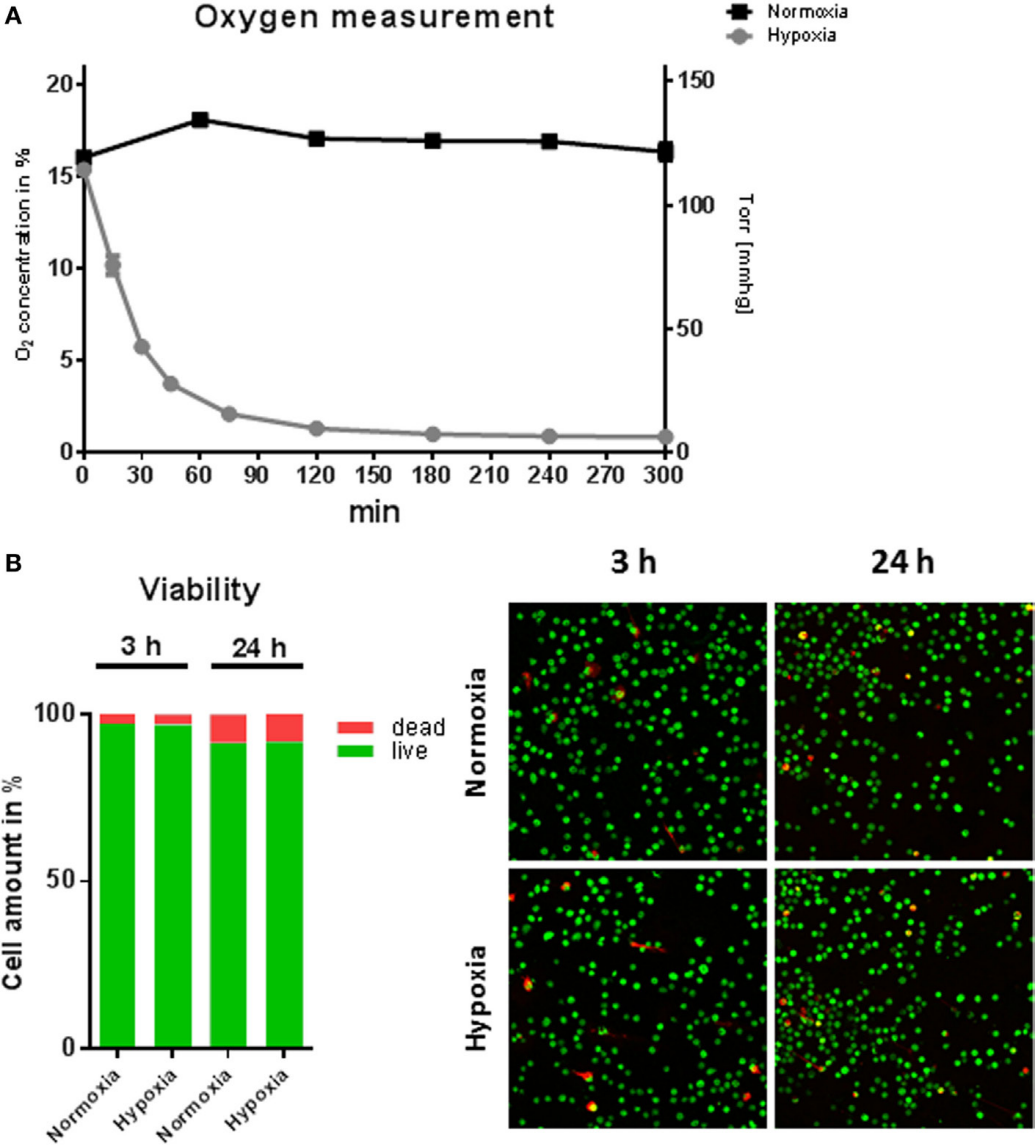
**Oxygen concentrations and viability of mast cells (MCs) under hypoxia**. **(A)** Oxygen concentrations in the media during culturing of MCs under hypoxia or normoxia. MCs were incubated for 5 h in normoxic and hypoxic environments. The dissolved oxygen concentration of the culture medium was measured using optical oxygen sensor spots (PreSens Precision Sensing GmbH) in intervals of 15 min to 1 h. Plotted values represent mean ± SEM of *n* = 3 experiments and are displayed as % oxygen on the left *y*-axis and mmHg oxygen on the right *y*-axis. **(B)** % viability of MCs (*n* = 6) after 3 and 24 h under hypoxia. Staining by a live/dead viability/cytotoxicity kit for mammalian cells (Invitrogen) indicates no differences in cell viability under hypoxia versus normoxia (red: dead cells; green: living cells).

To study the effect of hypoxic oxygen conditions on MC response to *S. aureus*, BMMCs were incubated either in a hypoxic glove box (7 mmHg, 1% O_2_) or in a regular CO_2_ incubator (159 mmHg, 21% O_2_). Initially, the oxygen content in the media was monitored under hypoxia versus normoxia over a time period of 5 h and cellular viability was examined. Under normoxia, the MC cultures maintained a constant oxygen level around 125 mmHg (16.9 ± 1.2% O_2_). The level is similar as shown for other suspension cultures, e.g., neutrophils (16), but distinctly less than shown for epithelial cells grown as attached monolayer (31). However, hypoxic incubation decreased the dissolved oxygen level in the culture media to less than 28 mmHg (3.7 ± 0.7% O_2_) within 45 min and resulted in a stable equilibrium lower than 7 mmHg (0.9 ± 0.2% O_2_) within 5 h (Figure 1A). The viability of MC was analyzed after 3 and 24 h cultivation at 1% oxygen using the LIVE/DEAD viability/cytotoxicity kit for mammalian cells (Invitrogen). Thereby, dead cells are separated from living cells by microscopic two-color discrimination. No differences could be found in cellular viability after 3 h incubation as well as after 24 h incubation under hypoxia compared to normoxia as depicted in Figure 1B.

### HIF-1α Level in MCs Increase after 24 h Hypoxia, but Not 3 h Hypoxia

**Figure 2:**
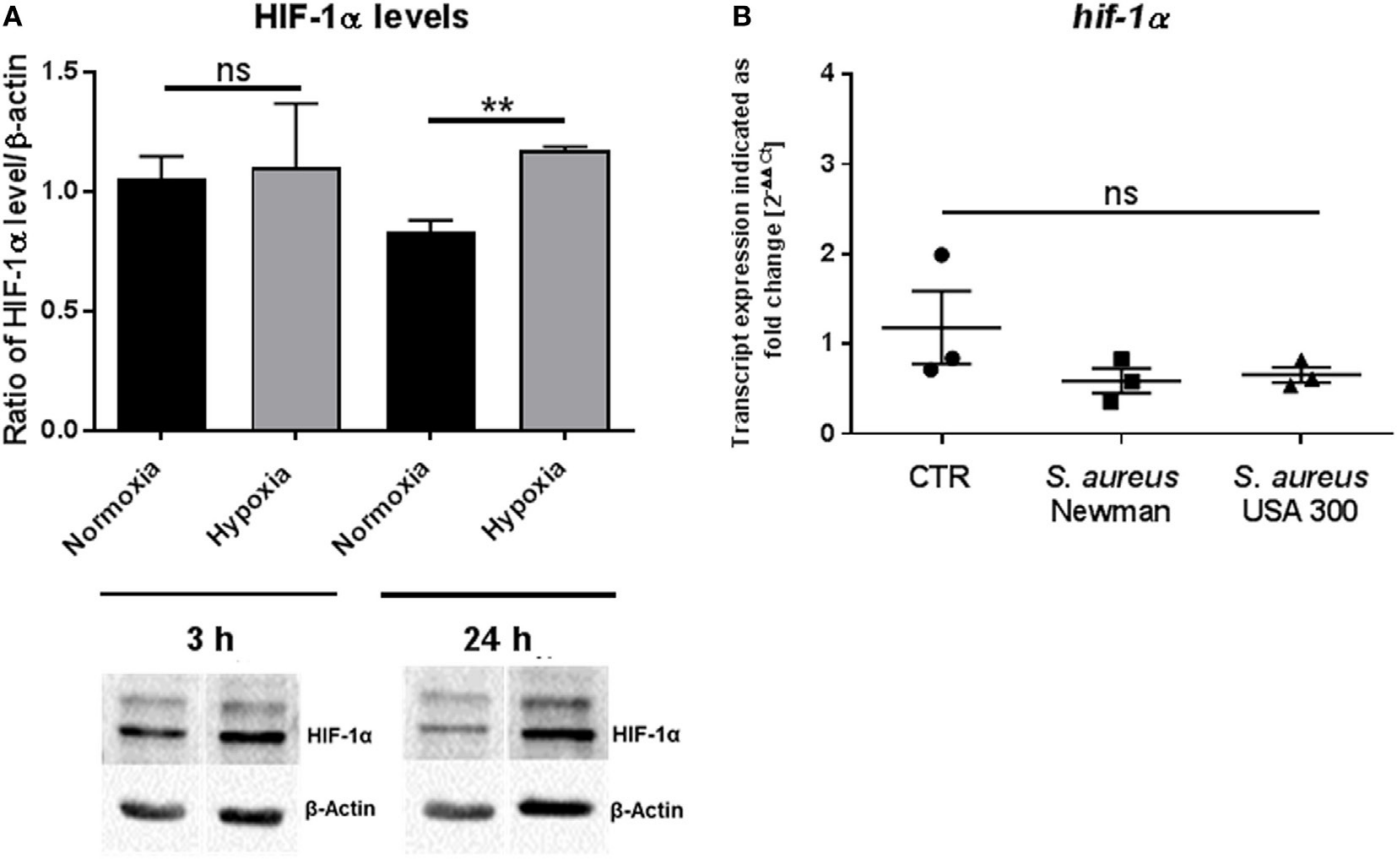
**HIF-1α protein levels**. **(A)** HIF-1α protein levels of whole cell lysates of mast cells (MCs) (*n* = 3) after 3 and 24 h under hypoxia and normoxia. HIF-1α was normalized to β-actin (ratio of HIF-1α/β-actin). Significantly more HIF-1α protein was detected after 24 h under hypoxia, not after 3 h. Representative Western blot: total cell extracts (75 μg) were separated by SDS/PAGE (10% gel) and transferred to nitrocellulose membranes. HIF-1α protein (120 kDa) was detected by the primary antibody against HIF-1α (rabbit anti-HIF-1α, Genetex, 2 μg/ml). β-actin (43 kDa) was used as a housekeeping gene (mouse anti-β actin, Santa Cruz). **(B)** Transcript expression of *hif-1α* under hypoxia compared to normoxia (*n* = 3 independent experiments, all PCR runs were performed twice, depicted are the means of each run). MCs were incubated under hypoxia or normoxia for 3 h and incubated with h.i. *Staphylococcus aureus* Newman/USA 300 at a MOI of 1 for 45 min before RNA was isolated. Data were normalized to the non-regulated housekeeping gene *rps9*. The *x*-fold changes of the values from samples incubated under hypoxia were calculated against the normoxic samples. The spontaneous *hif-1α* gene expression (CTR) as well as infected with h.i. *S. aureus* Newman/USA 300 under hypoxia compared to normoxia did not change.

The ubiquitous transcription factor HIF-1α is a key regulator of cell homeostasis and cellular adaptation to oxygen stress (13). To evaluate HIF-1α protein level under hypoxia in MCs after short-term hypoxia (3 h) as well as after long-term hypoxia (24 h), Western blot analysis was used. By evaluating the ratio of the HIF-1α band intensity versus the housekeeping β-actin, which was not significantly altered under normoxia versus hypoxia, we confirmed that HIF-1α was significantly stabilized in MCs after 24 h hypoxia but most importantly not after 3 h, when compared to its respective normoxic control (Figure 2A). By comparing the 3 h time point with the 24 h time point, no significant time-dependent change in HIF-1α protein level was detected. Furthermore, the transcript expression of *hif-1α* was evaluated by RT-qPCR at 3 h hypoxia and confirmed no change in response to hypoxia, *S. aureus* Newman, or *S. aureus* USA 300 (Figure 2B).

### Hypoxia Affects MCET Release and Bacterial Uptake

**Figure 3:**
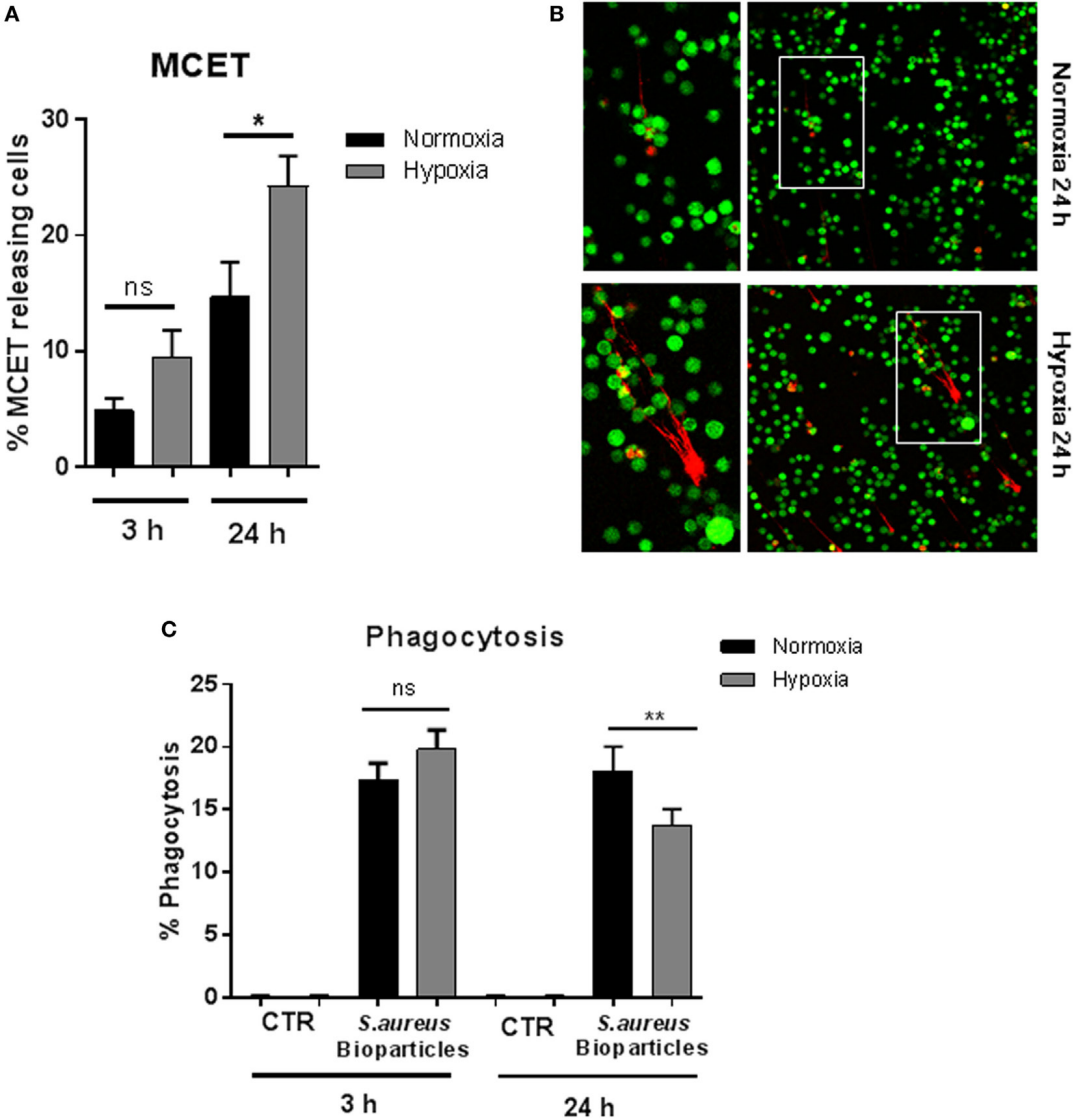
**MC extracellular trap (MCET) induction and phagocytosis of *Staphylococcus aureus* bioparticles**. **(A)** MCETs were visualized without fixation using the live/dead viability/cytotoxicity kit (Invitrogen) for mammalian cells. Significantly more MCETs are found after 24 h hypoxia versus normoxia, but not after 3 h. Results are shown from the analysis of *n* = 3 independent experiments, each with four individual images. **(B)** Representative fluorescence micrograph of MCET induction (red: dead cells/MCETs in a fiber like structure; green living cells). **(C)** Mast cells (MCs) (2 × 10^6^ cells/ml) were preincubated 3 or 24 h under hypoxia (37°C, 1% O_2_, 5% CO_2_) or normoxia (37°C, 21% O_2_, 5% CO_2_). Then phycoerythrin (PE)-labeled *S. aureus* (Wood strain, bioparticles; Sigma) at an MOI of 60 was incubated with MCs for 30 min under the respective oxygen condition. CTR represents uninfected control. The cells were washed with PBS and centrifuged to remove non-phagocytosed bacteria. PE-fluorescence was measured using a Beckman Coulter EPICS XL flow cytometer. The red fluorescence intensity per MC (% gated) was recorded and represents the mean relative phagocytosis of PE-labeled *S. aureus* per MC of *n* = 3 independent experiments.

Since it has already been shown that pharmacologically induced HIF-1α mediates MCET formation (30), we determined MCET release after 3 and 24 h under 1% oxygen compared to normoxia. The number of MCET-producing cells was significantly increased after 24 h under hypoxia, but not after short-term hypoxia (Figures 3A,B). Nevertheless, a tendency of increased MCET formation after 3 h under hypoxia was detectable, but this effect was not statistically significant.

To analyze bacterial uptake, MCs were incubated with fluorescent *S. aureus* bioparticles (Sigma), and the percentage of fluorescent MCs was determined after removal of free, non-associated bacteria to demonstrate the engulfment of *S. aureus* under hypoxia. As shown in Figure 3C, we observed that the percentage of phagocytosing MCs remained unchanged after preincubation for 3 h at 1% oxygen. After incubating MCs for 24 h under hypoxia (Figure 3C), the percentage of phagocytic cells under hypoxia decreased. MCs alone, without *S. aureus* bioparticles (CTR) used as negative control did not give any signal (Figure 3C).

### Hypoxia Modulates the Release of TNF-α

MCs reside in close proximity to blood vessels and contribute to recruitment of effector immune cells by releasing prestored TNF-α (43).

**Figure 4:**
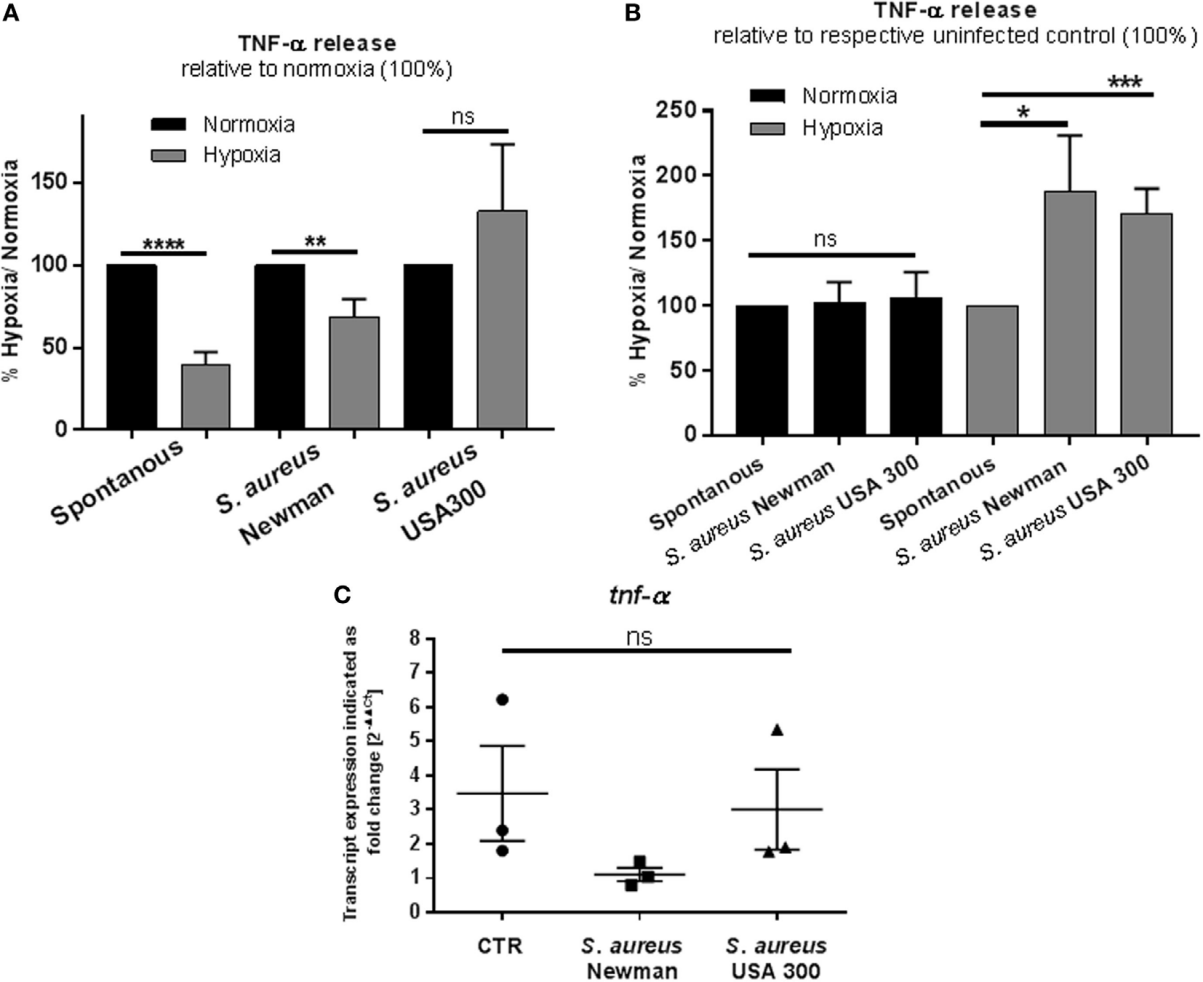
**TNF-α: release and transcript expression**. **(A)** TNF-α release of 1 × 10^6^ cell/ml was measured by a TNF-α ELISA (murine TNF-α ELISA development kit, PeproTech). Mast cells (MCs) were preincubated under hypoxia/normoxia for 3 h and infected with h.i. *Staphylococcus aureus* Newman/USA 300 at a MOI of 1 for 45 min. Comparing the release to the normoxic control (set to 100%), spontaneous and *S. aureus* Newman-induced TNF-α release is decreased under hypoxia (*n* = 3 independent experiments measured in triplicates). **(B)** TNF-α release in comparison to the untreated control (spontaneous release). The spontaneous release is set to 100% and compared to *S. aureus*-induced TNF-α release under the respective oxygen control. In comparison to its control, TNF-α release is not altered under normoxia. Under hypoxia, significantly more TNF-α is released, when stimulated with *S. aureus* Newman as well as USA 300 (*n* = 3 independent experiments measured in triplicates). **(C)** Transcript expression of *tnf-α* under hypoxia compared to normoxia (*n* = 3 independent experiments, all PCR runs were performed twice, depicted are the means of each run). MCs were incubated under hypoxia or normoxia for 3 h and incubated with h.i. *S. aureus* Newman/USA 300 at a MOI of 1 for 45 min before RNA was isolated. Data were normalized to the non-regulated housekeeping gene *rps9*. The *x*-fold changes of the values from samples incubated under hypoxia were calculated against the normoxic samples. The spontaneous *tnf-*α gene expression under hypoxia compared to normoxia is increased. Comparing the expression of *tnf-*α in response to *S. aureus* Newman, this induction phenomenon is abolished; the expression is decreased significantly in comparison to the spontaneous expression. No differences are observed for *S. aureus* USA 300-induced *tnf-α* expression.

To evaluate the early MC response mimicking an acute infection, the TNF-α release after 3 h cultivation under hypoxia was investigated and compared to normoxia. Therefore, spontaneous TNF-α release as well as its release in response to heat-inactivated *S. aureus* into the culture supernatant was measured by ELISA. Heat-inactivated bacteria were chosen to avoid any effect of hypoxia on bacterial growth or virulence. Interestingly, we found that the spontaneous as well as the *S. aureus* Newman-induced TNF-α release was decreased under hypoxia compared to normoxia (Figure 4A). Importantly, at the same time, the transcript expression of *tnf-α* under hypoxia compared to normoxia was increased in the CTR without infection (Figure 4C). Interestingly, calculating the TNF-α release relative to spontaneous release at respective oxygen conditions, MCs released significant higher amounts of TNF-α release upon stimulation with heat-killed *S. aureus* Newman as well as USA 300 at hypoxia compared to normoxia (Figure 4B). Calculating the transcript expression of *tnf-α* as response to *S. aureus* Newman relative to spontaneous transcript expression, respectively, no increased transcript level was detectable, confirming that the TNF-α release as response to infection is possibly based on prestored TNF-α (Figure 4C).

### Hypoxia Modulates the Release of Histamine

**Figure 5:**
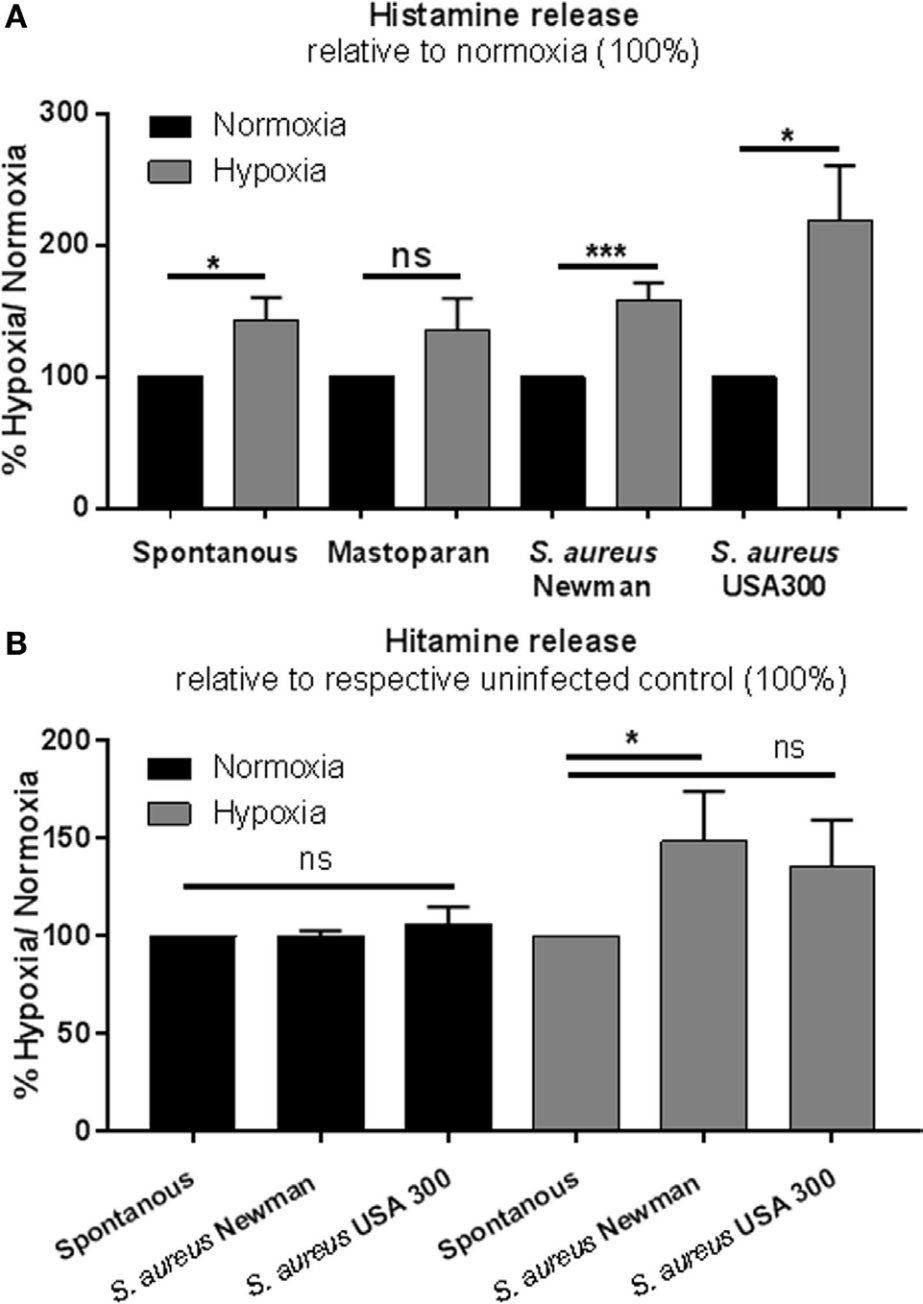
**Histamine release**. **(A)** Histamine release of 1 × 10^6^ cell/ml was measured by rpHPLC (*n* = 3 independent experiments measured in duplicates). Mast cells (MCs) were preincubated under hypoxia/normoxia for 3 h and incubated with a MOI 1 h.i. *Staphylococcus aureus* Newman and h.i. *S. aureus* USA 300, stimulated as a positive control with the MC-degranulating peptide mastoparan (50 μM Bachem, Heidelberg, Germany), and HBSS (spontaneous release) for 45 min. Comparing the release to the normoxic control (set to 100%), spontaneous *S. aureus* Newman and *S. aureus* USA 300-induced histamine release is increased under hypoxia. No differences in mastoparan-induced histamine release are observed between hypoxic and normoxic conditions. **(B)** Histamine release comparing the spontaneous release (set to 100%) and the *S. aureus-*induced histamine release under normoxic and hypoxic conditions (*n* = 3 independent experiments measured in duplicates). Histamine release under normoxia is not altered by *S. aureus*. Under hypoxia, significantly more histamine is released, when stimulated with *S. aureus* (significant for *S. aureus* Newman).

The release of histamine was measured by rpHPLC. Cells were incubated for 3 h at 1% oxygen and stimulated as described above. In contrast to TNF-α, the release of histamine was significantly elevated under low oxygen compared to normoxia (Figure 5A). In good correlation to the release of TNF-α in response to *S. aureus*, histamine release was also significantly increased under hypoxia, but not under normoxia (Figure 5B). These data indicate that MCs exhibit a more sensitive response to *S. aureus* under physiological hypoxic than under normoxic conditions.

### Lipid Alteration under Hypoxia

**Figure 6:**
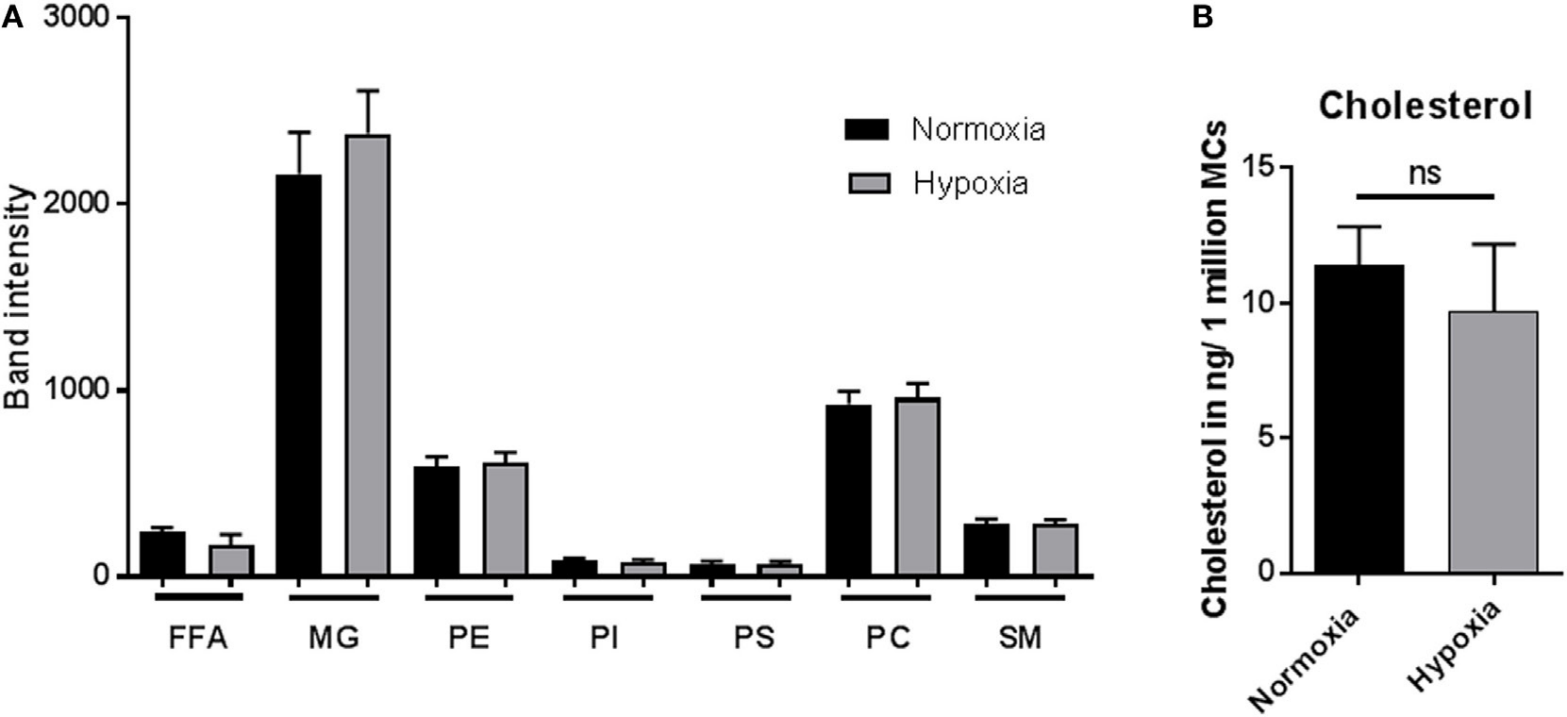
**Lipid alteration and cholesterol content**. **(A)** Mast cells (MCs) (3 × 10^6^ cells/ml) were incubated under hypoxia or normoxia for 3 h, and their lipid composition was analyzed by high performance thin layer chromatography (*n* = 3 independent experiments in duplicates) **(A)**: free fatty acids (FFAs), monoacylglycerols (MGs), phosphatidylethanolamine (PE), phosphatidylinositol (PI), phosphoserine (PS), phosphatidylcholine (PC), and sphingomyelin (SM). No differences could be observed comparing hypoxia and normoxia. **(B)** Cholesterol content (nanogram per 1 × 10^6^ MCs) was analyzed *via* HPLC (*n* = 4 independent experiments in duplicates). The cellular cholesterol level was not affected.

The lipid composition of a cell is well known to mediate its biological function (44). To identify if lipid alterations play a role in MC function under hypoxia, lipid composition was analyzed by HPTLC (Figure 6A) after 3 h incubation under hypoxia versus normoxia. The band intensities of FFAs, MGs, phosphatidylethanolamine (PE), phosphatidylinositol (PI), phosphoserine (PS), phosphatidylcholine (PC), and sphingomyelin (SM) were examined. No differences in band intensities of any of the tested lipids were found comparing hypoxia and normoxia (Figure 6A). To verify these results, the cellular cholesterol levels were quantified by HPLC. In accordance with the HPTLC data, the cholesterol levels, depicted as cholesterol in nanogram/one million cells, were also not altered under hypoxia compared to normoxia (Figure 6B).

### Microarray Transcriptome Analysis

**Figure 7:**
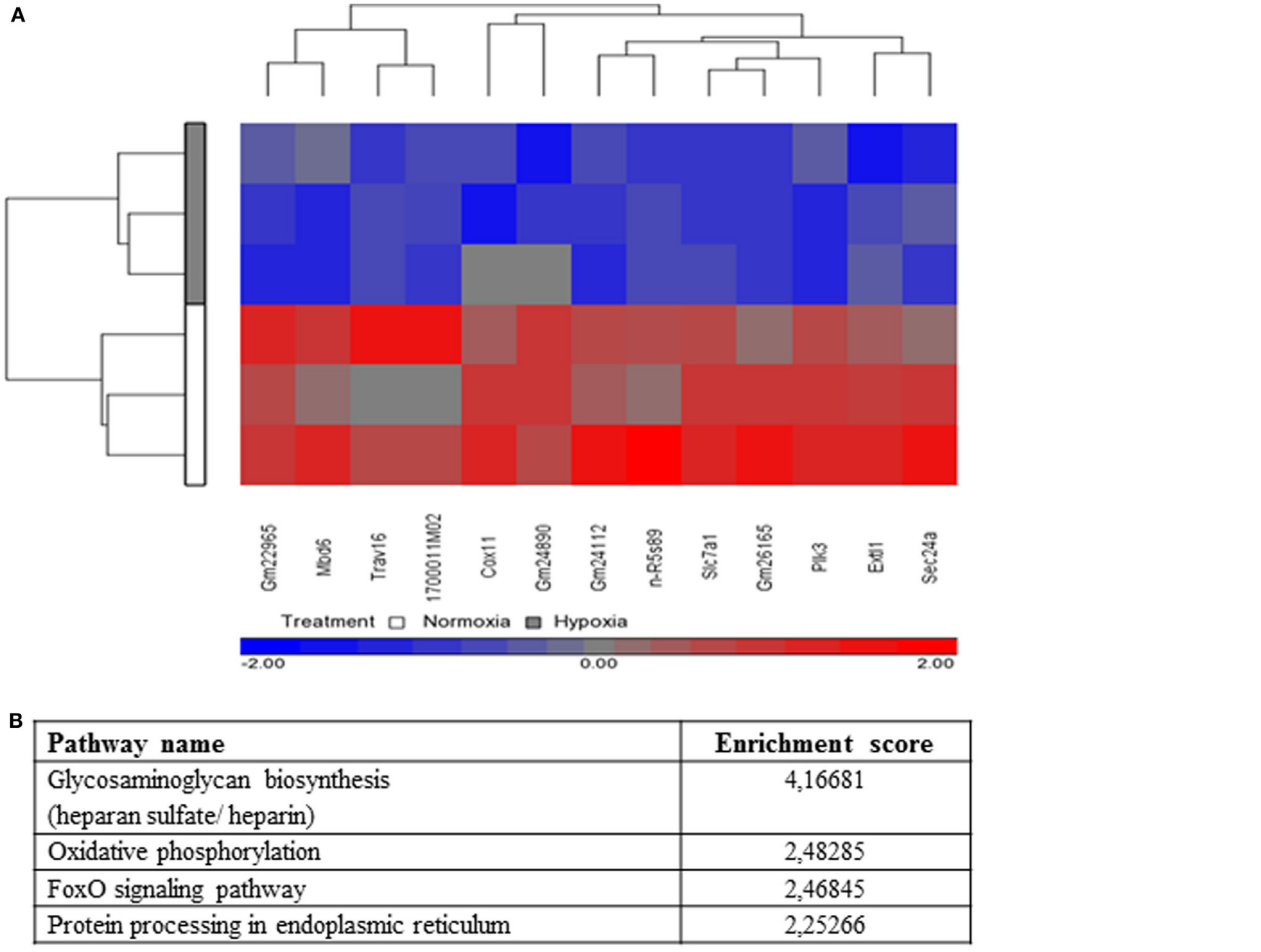

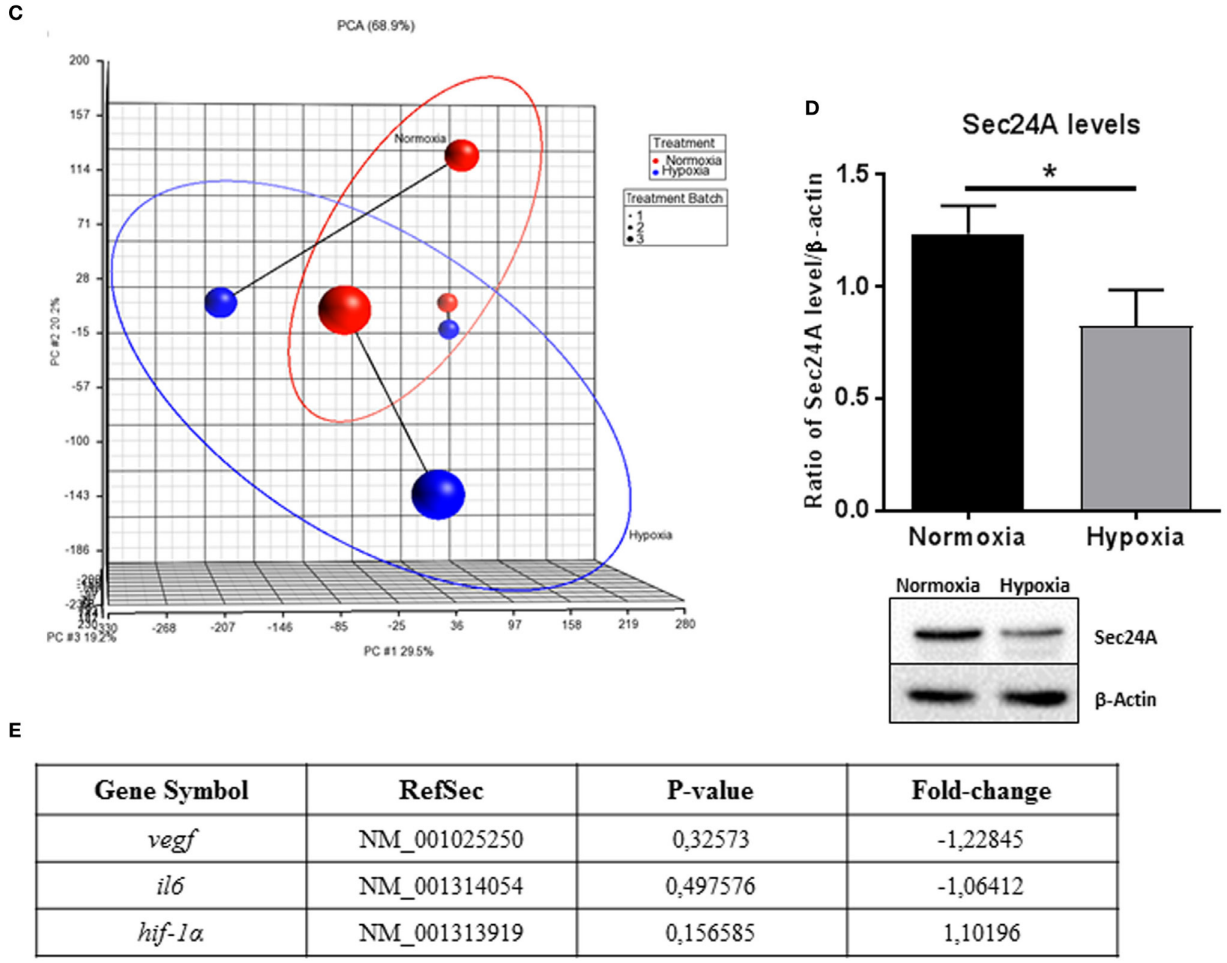
**Microarray expression analysis**. **(A)** Hierarchical clustering of genes with significantly different expression across hypoxia versus normoxia (change in hypoxia relative to normoxia with *p*-value <0.05, fold change > 1.3, or fold change < −1.3). Genes that are upregulated appear in red, and those that are downregulated appear in blue. **(B)** Selected top pathways from the Partek analysis sorted by the Enrichment Score (Fisher’s Exact test). **(C)** Principle component analysis (PCA) of microarray results (*n* = 3). The blue (hypoxic-treated cells) and red (normoxic-treated cells) oval dots represent linear combinations of the expression data, including relative expression value and variance. The PCA in PGS software examines three components of genes in different samples for those with similar or different expression profiles. PCA (Partek software) was used to compare the gene expression signatures of hypoxia with those of normoxia and connected to treatment Batch (1, 2, and 3). The percentage of overall variance is indicated for each PC and for the combination of all three PCs. The two ellipses show how the samples are grouped by treatment and line-connected by treatment batch. **(D)** Sec24A protein levels of whole cell lysates of mast cells (*n* = 3) after 3 h under hypoxia and normoxia. Sec24A was normalized to β-actin (ratio of Sec24A/β-actin). Significantly less Sec24A protein was detected after 3 h under hypoxia compared to normoxia. Differences between the two groups were analyzed by using a paired, two-tailed *t*-test. Representative western blot: total cell extracts (50 μg) were separated by SDS/PAGE (10% gel) and transferred onto PVDF membranes (Roth, Germany). Sec24A protein (66 kDa) was detected by the primary antibody against Sec24A (rabbit anti-Sec24A, Proteintech, 0.2 μg/ml). β-actin (43 kDa) was used as a housekeeping gene (mouse anti-β actin, Santa Cruz). **(E)** Microarray transcript expression analysis of *hif-1α* and its target genes *vegf* and *il-6*. No significant changes in gene expression are observable.

To understand cellular changes under short-term hypoxia on the transcriptional level, microarray transcriptome analysis was conducted. Statistical analysis of the transcript level revealed no significant upregulation of transcripts but a significant downregulation (depicted in blue) of the following 13 genes (Table 2) due to hypoxia compared to normoxia (Figure 7A).

**Table 2:** **Significant downregulated genes (hypoxia versus normoxia)**.

Gene symbol	RefSeq	*p*-Value	Fold-change
*1700011M0rik*	NR_073044	0.0321123	−1.3318
*cox11*	NM_199008	0.0332452	−1.32418
*extl1*	NM_019578	0.0221526	−1.33476
*gm22965*	ENSMUST00000157958	0.00417405	−1.31328
*gm24112*	ENSMUST00000179159	0.0111114	−1.50208
*gm24890*	ENSMUST00000104389	0.0219378	−1.31293
*gm26165*	ENSMUST00000083791	0.00779937	−1.56434
*mbd6*	NM_033072	0.0160515	−1.31043
*plk3*	NM_001313916	0.00348817	−1.33062
*sec24A*	XM_006534485	0.0159795	−1.37672
*slc7a1*	NM_001301424	0.00140855	−1.37927
*trav16*	OTTMUST00000035702	0.03533	−1.38341
*n-r5s89*	ENSMUST00000083532	0.035715	−1.31883

Significantly enriched pathways from the Partek pathway analysis (see Materials and Methods) sorted by the Enrichment Score (Fisher’s exact test) are depicted in Figure 7B. This analysis revealed that the significantly affected genes under hypoxia are involved in the glycosaminoglycan biosynthesis (heparan sulfate/heparin), the oxidative phosphorylation, the foxO signaling pathway, and the protein processing in endoplasmic reticulum.

By analyzing linear combinations of the expression data in general, including relative expression value and variance, all three independent samples exhibited an overall decreased transcript level under hypoxia compared to normoxia (Figure 7C).

One interesting candidate of the 13 significantly downregulated genes is *sec24a*, a member of the pre-budding complex coat protein complex II (COPII). To confirm the downregulation of *sec24a* on the protein level, western blot analysis was used (Figure 7D). By evaluating the ratio of the Sec24A band intensity versus the housekeeping β-actin, it was confirmed that the Sec24A protein level was significantly downregulated under hypoxia compared to normoxia (Figure 7D).

## Discussion

At sites of infection and inflammation, the oxygen concentration in the tissue decreases rapidly to hypoxic levels (eventually less than 1% O_2_) due to oxygen consumption by invading pathogens and infiltrating immune cells. Several studies suggest that the antimicrobial mechanisms of cells of the innate immune response differ significantly at hypoxic oxygen levels (15, 28) compared to 21% oxygen level normally used in tissue culture experiments. In this study, the antimicrobial activity of MCs under hypoxia compared to normoxia was investigated, with special focus on short-term response mimicking the acute phase of infection.

First, the drop of oxygen in the culture media of MCs under hypoxia and normoxia was recorded and indicated that the experimental settings applied by incubating MCs under hypoxia (1%) decrease the oxygen level and may reflect physiological oxygen conditions that occur in infected tissue (13). The viability of MCs did not differ neither after 3 h nor after 24 h under hypoxia compared to normoxia revealing that the majority of MCs survive even long-term hypoxia. These data are in good correlation to other reports showing that IL-6 secretion is increased in human MCs under hypoxia and that this autocrine produced IL-6 promotes MC survival, implicating that MCs are well adapted to the physiological oxygen level in the host tissue, and moreover under prolonged hypoxic conditions (45–48).

During the acute phase of an infection, a fast response of immune cells under hypoxic conditions is required. HIF-1α is a global regulator of the cellular response to oxygen stress. Nevertheless, it is reported that some cells such as Hela cells are able to react to acute hypoxia (2 h) in a HIF-independent manner by downregulating endocytosis (49). Here, we demonstrate that HIF-1α is significantly stabilized on the protein level after 24 h under hypoxia, but not after 3 h. This is the first evidence that during the early phase of infection, MCs do not respond to short-term hypoxia (3 h) *via* HIF-1α. In good correlation to the protein level, no altered transcript expression of HIF-target genes *vegf* (*p*-value: 0.32573/fold-change: −1.22845) or *il6* (*p*-value: 0.497576/fold-change: −1.06412) and also no change in transcript level of *hif-1α* itself (*p*-value: 0.156585/fold-change: −1.10196) were observed by microarray transcript analysis (Figure 7E). Importantly, when the infection establishes with severe prolonged hypoxic conditions, here characterized as long-term hypoxia (24 h), MCs adapt to hypoxia by stabilizing HIF-1α on protein level (Figure 2). These results confirm earlier findings of HIF-1α stabilization in neutrophils when short-term hypoxia did not alter the expression of *hif-1α* (16) and the HIF-1α protein level (50).

The repertoire of MCs’ antimicrobial activity includes the intracellular killing of pathogens after phagocytic uptake and the extracellular entrapment of pathogens by extracellular traps. Here, we show that phagocytosis is decreased after long term, however, not after short-term hypoxia. These findings correlate with the increased HIF-1α-dependent release of MCETs after long-term hypoxia. It has already been reported, for neutrophils, that an increase in NET formation comes along with a decrease in phagocytosis. Furthermore, when neutrophils undergo morphological changes to release NETs, phagocytic killing is decreased (51). This finding also seems to be true for MCs (2). Nonetheless, since the bacterial phagocytic efficiency of MCs is much less than professional phagocytic cells, the physiological relevance of this phenomenon is still unclear (3).

Mediator release is a major feature of MCs and of particular importance for the host immune defense upon infection. In our study, histamine release increased under hypoxia compared to normoxia and raised even more upon *S. aureus* stimulation, whereas the release of TNF-α was reduced under hypoxia in uninfected and *S. aureus* Newman-infected samples. The difference between the two bacterial *S. aureus* strains could be explained by the fact that both strains differ in their virulence potential, e.g., fibronectin-mediated adherence and invasion of the host cells (52): the Newman strain harbors truncated fibronectin-binding proteins, which limits adherence and invasion to host cells (52). But importantly, independent of the strain, the *S. aureus*-mediated changes in mediator release were more pronounced under hypoxia than under normoxia. Rocha-de-Souza et al. (53) showed that both alive and dead *S. aureus* trigger TNF-α release from cord blood-derived MCs in a time-dependent manner under normoxia (30 min to 6 h). This discrepancy to our data could be explained with differences in the MOI that we used in the experiments as well as the MC type. However, our findings emphasize indeed that MCs react with a lower amount of TNF-α, however, considerably more sensitive and rapidly under hypoxia than under normoxia. It has already been shown for human cord blood-derived MCs that 24 h hypoxia *per se* did not induce MC degranulation (45). In contrast to MCs, it is known for neutrophils that degranulation is increased under hypoxia in a HIF-independent manner, deploying harmful proteins and proteases to the extracellular milieu and increasing the possibility of tissue injury (54). The reduced TNF-α release in MCs under hypoxia, determined in our study, could have an immunomodulatory background to reduce an overwhelming inflammation by recruited immune cells.

Our results regarding histamine release are supported by the findings that high concentrations of histamine produced by residing MCs are found in areas of inflammation (55) in predominantly hypoxic microenvironments (13). The spontaneous histamine release under long-term hypoxia was already shown for HMC-1 cells and BMMCs in an anaerobic chamber (0.2% O_2_) (56). In a kinetic study, it was shown that significantly more histamine was secreted after 24 h under hypoxia in comparison to normoxia. A short-term effect could not be detected, but a tendency to more histamine under hypoxia is obvious in all those studies. Differences to our study could be explained by the different methods that were used. HPLC measurements used in this study are much more sensitive than colorimetric plate readouts used in previous publications.

Histamine is secreted by MCs within 10 min of stimulation and, therefore, one of the first reactions to antigen challenges, in contrast to TNF-α needing a minimum of 3 h to be released (57). It has been shown that histamine release during allergic reactions also exhibits anti-inflammatory effects through activated H2 receptors. Interestingly, histamine inhibits the TNF-α release in a concentration and time-dependent manner (57). The basal concentration in the human body is 8 × 10^−9^ M; a minimum concentration of 10^−12^ M was shown to significantly inhibit TNF-α release by MCs (57). Therefore, the authors discuss that histamine may be an important endogenous modulator of inflammatory and immune responses. These findings support our results that histamine is increased when TNF-α is decreased under hypoxia. Histamine may inhibit TNF-α release to avoid uncontrolled degranulation and thereby an acute overwhelming inflammation under hypoxia. Interestingly, in our study, HIF-1α as central transcriptional regulator of oxygen shortage was not altered on protein level indicating an HIF-1α-independent phenotype. For LAD2-MCs, it was shown that histamine release was not affected in HIF-1α knockdown cells (58) supporting our hypothesis that MCs adapt to short-term hypoxia in a HIF-1α-independent manner.

Lipids have been shown to play important roles in a wide range of cellular functions, e.g., signaling, ion exchange, autophagy, apoptosis, extracellular trap formation, antimicrobial functions, protein, and lipid trafficking (44, 59, 60). Especially, glycosphingolipids and cholesterol-enriched domains in cellular membranes, named lipid rafts, play an important role in cell homeostasis (61). Indeed, lipid rafts or raft components modulate many of the biological processes in MCs, such as degranulation and endocytosis and play a role in MC development and recruitment (62). Furthermore, for neutrophils, it has already been shown that the cholesterol level is significantly increased after 3 h incubation under hypoxia compared to normoxia at the same time when decreased spontaneous NET formation was detectable in control cells (16). We show here that the lipid composition was not significantly altered in MCs under short-term hypoxia. This lead to the assumption that lipid alterations may not play a major role in the early adaptation to hypoxia. However, since we only looked at certain lipid classes, additional metabolites may be altered due to fluctuating intracellular oxygen levels and enzyme activities. Therefore, future work will need to be conducted in this field.

To understand the cellular changes after short-term hypoxia (3 h) on the transcriptional level, microarray analysis was performed to give further insights. It could be observed that 13 genes were significantly downregulated under hypoxia. One interesting candidate is *sec24a*, a member of the pre-budding complex of COPII, which is responsible for the anterograde transport of proteins from the ER to the Golgi (63). The downregulation of Sec24A was additionally confirmed on the protein level (Figure 7D). These data lead to the suggestion that *de novo* synthesized proteins, which are crucial factors in the response to an acute infection, are partially retained in the ER under hypoxia. For macrophages, it is already known that cytokines such as IL-2, 3, 6, 10, 12, and TNF-α contain a signal peptide targeting them to the ER, where they are packaged into COPII-coated vesicles and delivered to the ER–Golgi intermediate compartment (ERGIC) (64). The downregulation of *sec24a* could, therefore, explain the upregulated expression of *tnf-α*, but its decreased release under hypoxia compared to normoxia. *De novo* synthesized TNF-α may retain in the ER without being efficiently transported to the Golgi for further modifications. These data are another hint that not only internalization *via* exocytosis is modified under hypoxia (49) but also intracellular transport and posttranslational modification of proteins. Certainly, further experiments are needed to verify this hypothesis.

However, the Partek pathway analysis (Figure 7B) revealed that the significantly affected genes under hypoxia are not only involved in protein processing of endoplasmic reticulum as Sec24A or in general oxygen metabolism as the oxidative phosphorylation but also in specific pathways as the glycosaminoglycan biosynthesis (heparan sulfate/heparin) and the foxO signaling pathway. These data are in good correlation with recent findings by Asplund et al. (65) who showed that 0.5% hypoxia increases macrophage motility, possibly by decreasing the heparan sulfate proteoglycan biosynthesis. Importantly, the authors show that HIF-1α is not involved in hypoxia-mediated changes of heparan sulfate proteoglycan biosynthesis, and the authors suggest that other oxygen sensitive transcription factors, e.g., NF-κB might be involved in that process (65). Interestingly, in 2008, a publication in Nature revealed a HIF-1α-independent regulation of oxygen stress by the transcriptional coactivator PGC-1α (peroxisome-proliferator-activated receptor-γ coactivator-1α), which is known to coactivate several transcription factors, including many members of FOXO (forkhead transcription factor O) or foxO signaling pathway (as found here in Partek pathway analysis Figure 7B) and nuclear receptor families (66). Thus, based on our data, we also hypothesize that similar HIF-1α-independent mechanisms might be involved in early adaptation of MCs to oxygen stress.

## Conclusion

Our data revealed that MCs adapt to long-term hypoxia by stabilizing HIF-1α and increasing the amount of MCETs and at the same time decreasing the amount of phagocytic active cells. During short-term hypoxia, reflecting an acute local infection, MCs do not adapt *via* stabilization of HIF-1α. MCs react by releasing a high amount of histamine under hypoxia after stimulation with heat-killed *S. aureus*. At the same time, TNF-α release is decreased under hypoxia compared to normoxia. Microarray transcriptome analysis unraveled a downregulation of *sec24a*, which is implicated in the COPII-mediated transport of TNF-α from the ER to the Golgi. We hypothesize that MCs adapt to short-term hypoxia in a HIF-1α-independent manner by downregulating the release of proinflammatory cytokines like TNF-α. Thereby, MCs may avoid uncontrolled degranulation, which could lead to an excessive immigration of immune cells, tissue inflammation, and cell damage (54), thereby orchestrating the overall immune response (8). Finally, our experiments on immune cell functions highlight the regulatory role of oxygen and the relevance of physiological oxygen levels in *in vitro* studies.

Thus, a better understanding of immune cell functions under physiologically relevant oxygen levels is of special importance as it will reveal new targets for novel therapeutic interventions and strengthen the understanding and subsequently the modulatory possibilities of the immune system.

## Ethics Statement

This study was carried out in accordance with the recommendations of Nds. Landesamt für Verbraucherschutz und Lebensmittelsicherheit. The protocol was approved by the local ethical commission of Lower Saxony.

## Author Contributions

MK-B, KB-H, HM, and HN: conceived and designed the experiments; HM, KB-H, GB, AE, WO, MS, and HF: performed the experiments; HM, KB-H, GB, AE, WO, and MS: analyzed the data; HM, KB-H, and MK-B: wrote the paper. All authors proofread the paper.

## Conflict of Interest Statement

The authors declare that the research was conducted in the absence of any commercial or financial relationships that could be construed as a potential conflict of interest.
